# Supplementary material for: Risk and Prognostic Factors in Very Old Patients with Sepsis Secondary to Community-Acquired Pneumonia
Source: J Clin Med. 2019 Jul 2;8(7):961. doi: 10.3390/jcm8070961 (PMC6678833; doi:10.3390/jcm8070961)
Supplement: Supplementary file 1 [file jcm-08-00961-s001.pdf]

1. Methods

1.1. Microbiological Diagnosis

We collected information on sputum samples in all cases and on pleural fluid, tracheobronchial aspirate, and bronchoalveolar lavage fluid when available. Sputum and blood samples were obtained for bacterial culture in the emergency department before administering antibiotics. Respiratory samples were subjected to Gram and Ziehl–Neelsen staining and processed for bacterial, fungal, and mycobacterial cultures. Nasopharyngeal swabs for respiratory virus detection and urine samples for *Streptococcus pneumoniae* and *Legionella pneumophila* antigen detection were obtained within 24 hours of hospital admission. Blood samples for serological testing of atypical pathogens and respiratory viruses were taken at admission and in the third and sixth weeks thereafter.

1.2. Statistical Analysis

Trends in associated factors were analyzed using the Mantel–Hansel test for categorical variables and linear regression for continuous variables.

Generalized linear model (GLM) analyses [1] were performed to determine the influence of the risk factors on 30-day mortality in overall population. Models were defined using a binomial probability distribution and a logit link function, using inverse probability of treatment weights (IPTWs) [2] to account for biases due to observed confounders. First, each risk factor was tested individually. Second, a propensity score (PS) for patients with sepsis were developed. The PS was determined, irrespective of the outcome, through a multivariate logistic regression to predict the influence of 15 predetermined variables on the presence of sepsis. Variables were chosen for inclusion in the PS calculation according to the methods of Brookhart et al [3] and included variables associated with sepsis and outcome (gender, smoking status, alcohol consumption, influenza vaccine, pneumococcal vaccine, previous inhaled corticosteroids, previous systemic corticosteroids, previous antibiotic in last week, chronic pulmonary disease, chronic cardiovascular disease, chronic renal disease, chronic liver disease, diabetes mellitus, neurological disease, and nursing-home resident). IPTW used the PS to form a weight. Finally, the weight, sepsis and the year of admission were incorporated in the multivariable weighted logistic regression model of 30-day mortality, including all risk factors which showed an association in the univariate analyses ($p < 0.10$), and calculated in a stepwise backward elimination procedure, dropping non-significant variables until no further improvement of the Akaike's information criterion was achieved [4]. We investigated the missing data patterns for covariates and assumed missing at random (MAR) conditioned in the covariates. We used multiple imputation [5] to generate 5 datasets to evaluate the prediction performance for the sepsis and 30-day mortality. The model for multiple imputations included all covariates of the risk models as well as sepsis, and 30-day mortality. For simplicity, for the performance evaluation we filled in missing values with the first set of imputed values from the multiple imputations.

2. Results

2.1. Overall Population

The study population comprised 1238 very old patients with CAP, for whom the mean (SD) age was 86 (5) years and 686 (56%) were males. Also, 227 (18%) were nursing home residents, 985 (80%) presented one or more chronic comorbidities, 395 (32%) had a microbiological diagnosis, and 136 (11%) were admitted to ICU.

An etiologic diagnosis was achieved in 395 cases (32%). Blood culture was performed in 809 cases (65%), urinary antigen test for pneumococcus in 828 cases (67%), sputum culture in 436 (35%), nasopharyngeal swab in 427 cases (34%), bronchoalveolar lavage in 79 cases (6%) and culture of pleural fluid in 76 cases (6%).

Among the patients with a known etiology, the three most frequent causes of CAP were *S. pneumoniae* (170 patients (43%)), respiratory viruses (68 patients (17%)), and polymicrobial infection

(41 patients (10%)). The most frequent combination of pathogens in the 41 polymicrobial infections were: *S. pneumoniae* plus respiratory viruses (39%), *S. pneumoniae* plus *H. influenzae* (10%), and respiratory viruses plus *P. aeruginosa* (5%).

In the overall population, 455 patients (37%) were treated with a single antibiotic, with fluoroquinolones and β-lactams being the most commonly used monotherapies. We treated 769 patients (63%) with combination therapy, with the most frequent combinations being a β-lactam plus a macrolide (360 patients (29%)) or a β-lactam plus a fluoroquinolone (297 patients (24%)).

2.2. Comparison between Old (65–79 years) and Very Old (≥80 years) Patients

We compared patients aged 65–79 years (the old) and patients aged ≥80 years (the very old). The main characteristics and clinical outcomes by age groups are presented in the online data (Table S1). Focusing on statistically significant differences, we observed that very old patients were less frequently males, current smokers, and alcohol consumers. They were also less likely to have received previous inhaled or systemic corticosteroid, but were more likely to have received influenza vaccination. In addition, the very old group presented a higher proportion of neurological and chronic renal diseases, but a lower proportion of chronic respiratory and liver diseases. The rate of nursing home residents was also higher among very old patients. The uncommon presentation of pneumonia was frequent in very old patients: compared with patients aged 65–79 years, they presented a lower rate of fever, cough, pleuritic pain and a higher frequency of altered mental status at admission. Also, this uncommon presentation of pneumonia was observed in the subgroup of patient with and without sepsis. Very old patients also experienced severe CAP at a higher frequency. Although there was no significant difference in the percentage of sepsis between the two groups, the rates of septic shock and ICU admission were significantly higher in the old group. Notably, in-hospital mortality, ICU mortality, 30-day mortality, and 1-year mortality were significantly higher in the very old group compared with the old group.

2.3. Comparison of Very Old Patients from Home and Nursing Homes Residents

A total of 227 patients (19%) were nursing home residents. Compared with patients from home, nursing home residents were significantly older and more frequently females, but were less frequently smokers and alcohol consumers. They presented a significantly higher rate of neurological disease, but lower rates of chronic respiratory disease and diabetes mellitus. Severe CAP was more frequent in the nursing home group, but the groups had comparable sepsis presentations. The main characteristics of the two groups, including the main clinical outcomes, are presented in Table S2.

2.4. Very Old ICU and Non-ICU Patients

A total of 136 patients (11%) were admitted to ICU during the study period. Compared to those not admitted to ICU, these patients were significantly younger, more frequently male, and less frequently nursing home residents. They presented a significantly lower rate of neurological disease but a higher rate of diabetes mellitus. Severe CAP was more common in ICU patients. The percentages of sepsis and septic shock were significantly higher in the ICU group. Similarly, the length of stay and the in-hospital, 30-day, and 1-year mortality rates were significantly higher in the ICU group. The main characteristics of the ICU and non-ICU groups are presented in Table S3.

2.5. Changes in Sepsis Over Time in Very Old Patients

To investigate the changes in sepsis presentation over time, we divided the study population into four cohorts by period: 2005–2007, 2008–2010, 2011–2013, and 2014–2017. This revealed that the prevalence of sepsis in very old patients did not change significantly over time ($p = 0.051$), with rates ranging from 77% in the first period to 75% in the last period (Figure S1).

Table S1. Characteristics and clinical outcomes by age group.

Variable	Age Group		
	**65–79 y (*N* = 975)**	**≥80 y (*N* = 1238)**	***p*-Value**
Age, mean (SD), y	73 (4)	86 (5)	<0.001
Male sex, *n* (%)	625 (64)	686 (56)	<0.001
Current smoker, *n* (%)	141 (15)	55 (5)	<0.001
Current alcohol consumer, *n* (%)	139 (14)	76 (6)	<0.001
Previous antibiotic in last week, *n* (%)	220 (24)	288 (25)	0.45
Influenza vaccine, *n* (%)	503 (57)	652 (64)	0.004
Pneumococcal vaccine, *n* (%)	212 (24)	257 (25)	0.67
Previous inhaled corticosteroids, *n* (%)	291 (30)	247 (21)	<0.001
Previous systemic corticosteroids, *n* (%)	65 (7)	55 (5)	0.022
Previous episode of pneumonia (1 y), *n* (%)	151 (16)	164 (14)	0.24
Fever, *n* (%)	717 (75)	800 (66)	<0.001
Pleuritic pain, *n* (%)	325 (34)	268 (23)	<0.001
Dyspnea, *n* (%)	709 (74)	923 (77)	0.13
Cough, *n* (%)	730 (76)	873 (73)	0.058
Altered mental status, *n* (%)	216 (22)	359 (30)	<0.001
Comorbidities, *n* (%) [a]	764 (79)	985 (80)	0.37
Chronic respiratory disease [b]	486 (51)	471 (39)	<0.001
Chronic cardiovascular disease	180 (19)	258 (21)	0.14
Diabetes mellitus	270 (28)	311 (26)	0.19
Neurological disease	167 (18)	389 (32)	<0.001
Chronic renal disease	80 (8)	135 (11)	0.031
Chronic liver disease	48 (5)	37 (3)	0.020
Nursing home, *n* (%)	57 (6)	227 (18)	<0.001
PSI score, median (IQR)	105 (86; 125)	122 (102; 143)	<0.001
PSI risk class IV–V, *n* (%) [c]	430 (67)	584 (89)	<0.001
Severe CAP, *n* (%)	238 (31)	309 (37)	0.005
Bacteremia, *n* (%) [d]	75 (10)	98 (12)	0.23
Pleural effusion, *n* (%)	122 (13)	152 (13)	0.96
Multilobar, *n* (%)	229 (23)	277 (22)	0.54
Sepsis, *n* (%)	703 (72)	880 (71)	0.60
Septic shock, *n* (%)	72 (8)	61 (5)	0.021
Length of hospital stay, median (IQR), days	8 (6; 13)	8 (6; 12)	0.51
ICU admission, *n* (%)	245 (25)	136 (11)	<0.001
ICU mortality, *n* (%) [e]	16 (7)	21 (15)	0.005
Mechanical ventilation, *n* (%) [f]			<0.001
No ventilation	687 (84)	819 (92)	<0.001
Non-invasive	64 (8)	39 (4)	0.003
Invasive	71 (9)	36 (4)	<0.001
In-hospital mortality, *n* (%)	65 (7)	161 (13)	<0.001
30-days mortality, *n* (%)	57 (6)	174 (14)	<0.001
1-year mortality, *n* (%)	89 (9)	246 (20)	<0.001
Appropriate empiric treatment, *n* (%)	732 (95)	1156 (96)	0.27

Abbreviations: CAP, community-acquired pneumonia; ICU, intensive care unit; IQR, interquartile range; PSI, pneumonia severity index. Percentages calculated on non-missing data. [a] May have >1 comorbid condition. [b] Other respiratory diseases include sequelae of pulmonary tuberculosis, pulmonary hypertension, and interstitial lung disease. [c] Stratified according to 30-day mortality risk for CAP: classes I–III (≤90 points) have low mortality risk and classes IV–V (>90 points) have the highest mortality risk. [d] 737 patients in the 65–79 y group and 809 patients in the ≥80 y group were used to calculate the percentages. [e] 245 patients in the 65–79 y group and 136 patients in the ≥80 y group were used to calculate the percentages. [f] Patients who initially received non-invasive ventilation but subsequently needed intubation were included in the invasive mechanical ventilation group.

Table S2. Characteristics and clinical outcomes of very old patients from nursing home and own home environments.

Variable	Own Home (N = 1002)	Nursing Home (N = 227)	p-Value
Age, mean (SD), y	86 (5)	87 (5)	<0.001
Male sex, *n* (%)	587 (59)	91 (40)	<0.001
Current smoker, *n* (%)	51 (5)	3 (1)	0.015
Current alcohol consumer, *n* (%)	69 (7)	7 (3)	0.037
Previous antibiotic in last week, *n* (%)	235 (25)	53 (26)	0.66
Influenza vaccine, *n* (%)	552 (64)	99 (63)	0.81
Pneumococcal vaccine, *n* (%)	224 (26)	32 (21)	0.17
Previous inhaled corticosteroids, *n* (%)	211 (22)	36 (17)	0.11
Previous systemic corticosteroids, *n* (%)	40 (4)	15 (7)	0.079
Previous episode of pneumonia (1 y), *n* (%)	133 (14)	31 (16)	0.48
Comorbidities, *n* (%) [a]	783 (78)	197 (88)	0.002
Chronic respiratory disease [b]	400 (41)	67 (31)	0.004
Chronic cardiovascular disease	201 (20)	56 (25)	0.10
Diabetes mellitus	264 (27)	44 (20)	0.039
Neurological disease	260 (27)	127 (58)	<0.001
Chronic renal disease	113 (11)	20 (9)	0.30
Chronic liver disease	28 (3)	8 (4)	0.52
PSI score, median (IQR)	119 (100; 141)	133 (120; 155)	<0.001
PSI risk class IV–V, *n* (%) [c]	485 (88)	99 (99)	0.001
Severe CAP, *n* (%)	244 (35)	61 (48)	0.004
Sepsis, *n* (%)	719 (72)	156 (69)	0.36
Septic shock, *n* (%)	50 (5)	8 (4)	0.36
Length of hospital stay, median (IQR), days	8 (6; 13)	8 (5; 11)	0.19
ICU admission, *n* (%)	123 (12)	10 (4)	0.001
ICU mortality, *n* (%) [d]	17 (14)	3 (30)	0.17
In-hospital mortality, *n* (%)	109 (11)	49 (22)	<0.001
30-day mortality, *n* (%)	119 (12)	52 (23)	<0.001
1-year mortality, *n* (%)	169 (17)	73 (32)	<0.001

Abbreviations: CAP, community-acquired pneumonia; ICU, intensive care unit; IQR, interquartile range; PSI, pneumonia severity index. Percentages calculated on non-missing data. [a] May have >1 comorbid condition. [b] Other respiratory diseases include sequelae of pulmonary tuberculosis, pulmonary hypertension, and interstitial lung disease. [c] Stratified according to 30-day mortality risk for CAP: classes I–III (≤90 points) have low mortality risk and classes IV–V (>90 points) have the highest mortality risk. [d] 123 patients in the own home group and 10 patients in the nursing home group were used to calculate the percentages.

Table S3. Characteristics and clinical outcomes of very old ICU and non-ICU patients.

Variable	Non-ICU (N = 1102)	ICU (N = 136)	p-Value
Age, mean (SD), y	86 (5)	84 (3)	<0.001
Male sex, n (%)	595 (54)	91 (67)	0.005
Current smoker, n (%)	46 (4)	9 (7)	0.19
Current alcohol consumer, n (%)	66 (6)	10 (8)	0.51
Previous antibiotic in last week, n (%)	261 (25)	27 (22)	0.41
Influenza vaccine, n (%)	585 (64)	67 (61)	0.63
Pneumococcal vaccine, n (%)	228 (25)	29 (26)	0.75
Previous inhaled corticosteroids, n (%)	218 (20)	29 (22)	0.72
Previous systemic corticosteroids, n (%)	46 (4)	9 (7)	0.19
Previous episode of pneumonia (1 y), n (%)	149 (14)	15 (12)	0.43
Nursing home, n (%)	217 (20)	10 (8)	0.001
Comorbidities, n (%) [a]	877 (80)	108 (80)	0.98
Chronic respiratory disease [b]	411 (39)	60 (45)	0.13
Chronic cardiovascular disease	235 (22)	23 (17)	0.27
Diabetes mellitus	267 (25)	44 (33)	0.049
Neurological disease	366 (34)	23 (18)	0.001
Chronic renal disease	116 (11)	19 (14)	0.21
Chronic liver disease	32 (3)	5 (4)	0.59
PSI score, median (IQR)	120 (101; 142)	135 (120; 154)	<0.001
PSI risk class IV–V, n (%) [c]	500 (89)	84 (94)	0.10
Severe CAP, n (%)	217 (31)	92 (72)	<0.001
Sepsis, n (%)	761 (69)	119 (88)	<0.001
Septic shock, n (%)	20 (2)	41 (31)	<0.001
Length of hospital stay, median (IQR), days	8 (6; 12)	12 (8; 19)	<0.001
ICU mortality, n (%)	-	21 (15)	-
In-hospital mortality, n (%)	125 (11)	37 (27)	<0.001
30-day mortality, n (%)	141 (13)	33 (24)	<0.001
1-year mortality, n (%)	203 (19)	43 (33)	<0.001

Abbreviations: CAP, community-acquired pneumonia; ICU, intensive care unit; IQR, interquartile range; PSI, pneumonia severity index. Percentages calculated on non-missing data. [a] May have >1 comorbid condition. [b] Other respiratory diseases include sequelae of pulmonary tuberculosis, pulmonary hypertension, and interstitial lung disease. [c] Stratified according to 30-day mortality risk for CAP: classes I–III (≤90 points) have low mortality risk and classes IV–V (>90 points) have the highest mortality risk.

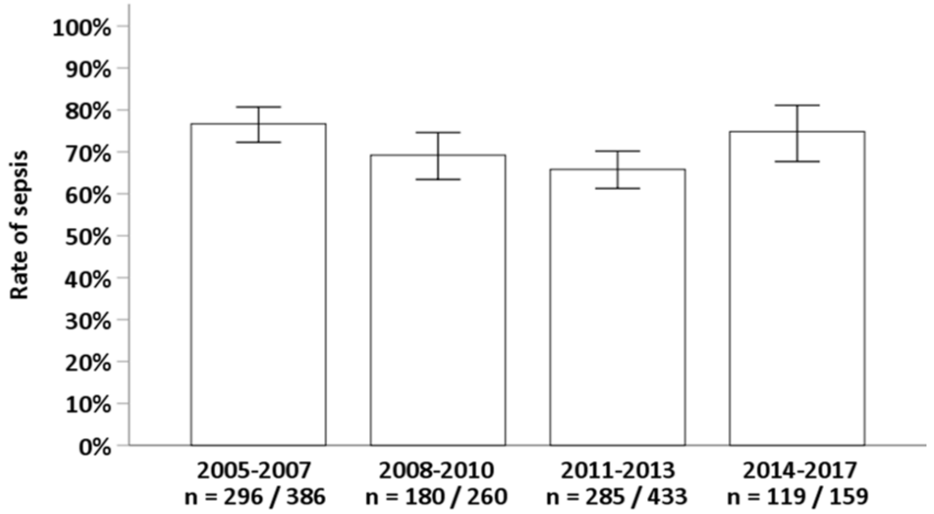

Figure S1. Rate of Sepsis by Period.

2.6. Septic Patients with Septic Shock

Of the 856 patients with sepsis, 61 (7%) developed septic shock (these patients were significantly younger than the group without septic shock). In-hospital mortality, ICU mortality, 30-day, and 1-year mortality were significantly higher in the patients with septic shock than in those without septic shock. The main characteristics of these groups are presented in Table S4.

Table S4. Characteristics and clinical outcomes of very old patients with and without septic shock.

**Variable**	**Without Septic Shock (*N* = 795)**	**With Septic Shock (*N* = 61)**	***p*-Value**
Age, mean (SD), y	86 (5)	84 (4)	<0.001
Male sex, *n* (%)	474 (60)	43 (70)	0.097
Current smoker, *n* (%)	42 (5)	3 (5)	>0.99
Current alcohol consumer, *n* (%)	51 (6)	4 (7)	0.79
Previous antibiotic in last week, *n* (%)	176 (23)	9 (17)	0.32
Influenza vaccine, *n* (%)	434 (64)	28 (61)	0.65
Pneumococcal vaccine, *n* (%)	167 (25)	16 (34)	0.15
Previous inhaled corticosteroids, *n* (%)	173 (22)	10 (17)	0.31
Previous systemic corticosteroids, *n* (%)	36 (5)	6 (10)	0.11
Previous episode of pneumonia (1 y), *n* (%)	126 (17)	5 (9)	0.16
Nursing home, *n* (%)	143 (18)	8 (14)	0.42
Comorbidities, *n* (%) [a]	640 (81)	50 (83)	0.59
Chronic respiratory disease [b]	310 (40)	27 (47)	0.31
Chronic cardiovascular disease	176 (22)	12 (20)	0.74
Diabetes mellitus	210 (27)	22 (37)	0.095
Neurological disease	247 (31)	12 (22)	0.16
Chronic renal disease	99 (13)	12 (20)	0.096
Chronic liver disease	21 (3)	3 (5)	0.23
PSI score, median (IQR)	122 (103; 143)	152 (141; 184)	<0.001
PSI risk class IV–V, *n* (%) [c]	468 (91)	33 (100)	0.10
Severe CAP, *n* (%)	221 (39)	61 (100)	<0.001
Length of hospital stay, median (IQR), days	8 (6; 13)	11.5 (7; 20)	0.001
ICU admission, *n* (%)	75 (9)	41 (67)	<0.001
ICU mortality, *n* (%) [d]	7 (9)	12 (29)	0.006
In-hospital mortality, *n* (%)	99 (13)	24 (39)	<0.001
30-day mortality, *n* (%)	104 (13)	24 (39)	<0.001
1-year mortality, *n* (%)	154 (20)	29 (48)	<0.001

Abbreviations: CAP, community-acquired pneumonia; ICU, intensive care unit; IQR, interquartile range; PSI, pneumonia severity index. Percentages calculated on non-missing data. [a] May have >1 comorbid condition. [b] Other respiratory diseases include sequelae of pulmonary tuberculosis, pulmonary hypertension, and interstitial lung disease. [c] Stratified according to 30-day mortality risk for CAP: classes I–III (≤90 points) have low mortality risk and classes IV–V (>90 points) have the highest mortality risk. [d] 75 patients in the non-septic shock group and 41 patients in the septic shock group were used to calculate the percentages.

References:

1. McCullagh, P.; Nelder, J.A. Generalized Linear Models, 2nd ed. CRC Press: Boca Raton, FL, USA, 1989.
2. Thoemmes, F.; Ong, A.D. A Primer on Inverse Probability of Treatment Weighting and Marginal Structural Models. *Emerging Adulthood* **2016**, *4*, 40–59.
3. Brookhart, M.A.; Schneeweiss, S.; Rothman, K.J.; Glynn, R.J.; Avorn, J.; Stürmer, T. Variable selection for propensity score models. *Am. J. Epidemiol.* **2006**, *163*, 149–1156.
4. Multivariate Statistical Modelling Based on Generalized Linear. Available online: https://www.springer.com/us/book/9780387951874 (accessed on 13 November 2018)

5. Sterne, J.A.; White, I.R.; Carlin, J.B.; Spratt, M.; Royston, P.; Kenward, M.G.; Wood, A.M.; Carpenter, J.R. Multiple imputation for missing data in epidemiological and clinical research: potential and pitfalls. *BMJ* **2009**, *338*, b2393.
